# Pharmacokinetics and Metabolomic Mechanism of the Diuretic Effects of Barbatic Acid from *Pyrrosia petiolosa* (Christ) Ching

**DOI:** 10.3390/biology15070521

**Published:** 2026-03-25

**Authors:** Shanshan Liang, Minjie Zhang, Juan Xue, Tianqiong Lang, Guoyong Luo, Yan Zhang, Xiang Yu, Wude Yang

**Affiliations:** 1 College of Pharmacy, Guizhou University of Traditional Chinese Medicine, Guiyang 550025, China; liangshanshan070@gzy.edu.cn (S.L.);; 2 Provincial Inheritance Base of Traditional Chinese Medicine Processing, National Administration of Traditional Chinese Medicine, Guizhou University of Traditional Chinese Medicine, Guiyang 550025, China

**Keywords:** Barbatic acid, *Pyrrosia petiolosa* (Christ) Ching, diuresis, pharmacokinetics, metabolomics

## Abstract

Barbatic acid (BA), a phenolic compound isolated from *Pyrrosia petiolosa* (Christ) Ching, was investigated for its diuretic effects and underlying mechanisms following oral administration in rats using UPLC-MS/MS-based metabolomics and pharmacokinetics. In a water-loaded rat model, BA (28 and 56 mg/kg) significantly increased 6 h urine output (1.5-fold vs. model, *p* < 0.01) and promoted urinary excretion of Na^+^, K^+^, and Cl^−^ (1.1–1.4-fold, *p* < 0.05–0.01). Metabolomic analysis revealed that BA modulates amino acid metabolism pathways, including cysteine and methionine metabolism (impact score 0.16), tyrosine metabolism (impact score 0.10), histidine metabolism (impact score 0.12), taurine hypotaurine metabolismand (impact score 0.43), and phenylalanine metabolism (impact score 0.14). Pharmacokinetic evaluation showed dose-dependent half-lives of 5.88, 5.23, and 2.61 h at 28, 56, and 112 mg/kg, respectively, with Cmax and AUC increasing proportionally with dose (r^2^ > 0.99). These findings provide the first integrated evidence supporting BA as a potential novel diuretic agent with a mechanism involving amino acid metabolism regulation.

## Simple Summary

This study provides the first in vivo investigation of barbatic acid (BA) from *Pyrrosia petiolosa* as a novel diuretic. In water-loaded rats, BA significantly increased urine output and promoted Na^+^, K^+^, and Cl^−^ excretion. Metabolomics revealed that BA modulates multiple amino acid metabolism pathways (e.g., taurine, histidine, tyrosine). Pharmacokinetics showed dose-dependent half-lives (2.6–5.9 h) and slow clearance. These findings highlight BA’s potential as a diuretic with a distinctive metabolic profile, warranting further research.

## 1. Introduction

*Pyrrosia petiolosa* (Christ) Ching (PPC), a fern traditionally used for its diuretic and lithagogue properties [1], has demonstrated clinical efficacy in urinary disorders [2,3]. Phytochemical investigations have identified phenolic acids as major bioactive constituents of PPC [4], which possess multiple biological functions including diuretic effects [5,6].

Barbatic acid (BA), a phenolic acid originally identified in lichens and subsequently isolated from the petroleum ether fraction of PPC by our group [7,8], has been shown to possess diuretic activity in MDCK cell-based assays [9]. These findings suggested its potential as a novel diuretic agent [10,11].

While BA exhibits no evident cytotoxicity in human hepatocytes [12] or peripheral blood mononuclear cells [13], and a synthetic route has been established to increase its yield [14], previous studies have only predicted its diuretic potential through in silico docking [5] and in vitro assays [10]. Building on our previous work, which identified BA from *P. petiolosa* and demonstrated its diuretic potential in vitro [5,10], the present study aimed to investigate the in vivo diuretic effect of BA and its underlying metabolomic and pharmacokinetic mechanisms. Specifically, no data are available on how BA modulates endogenous metabolism in vivo to promote diuresis, nor on its absorption, distribution, and elimination in living organisms. Based on our preliminary metabolomic findings and the known role of amino acid metabolism in renal function, we hypothesized that BA induces diuresis by modulating amino acid metabolism pathways thereby promoting electrolyte excretion and altering urinary uric acid levels. Therefore, this study employed UPLC-MS/MS-based metabolomics and pharmacokinetic analysis to investigate, for the first time, the integrated in vivo mechanism of BA—linking its diuretic effect to alterations in the metabolic network—and to characterize its bioavailability in rats. These findings provide the first direct evidence supporting BA’s development as a novel diuretic agent and offer a systems-level perspective on its mechanism of action.

The diuretic effect of BA, as demonstrated by increased urine output, electrolyte excretion, and modulation of amino acid metabolism, provides a scientific basis for understanding the traditional use of *Pyrrosia petiolosa* in TCM for ‘clearing heat and removing dampness’. By integrating metabolomics and pharmacokinetics, this study offers a paradigm for exploring the pharmacodynamic material basis of TCM and contributes to its modernization through contemporary pharmacological approaches.

## 2. Materials and Methods

### 2.1. Chemicals and Reagents

Barbatic acid (with a purity more than 98%) was prepared in the laboratory. Emodin (IS, with a purity more than 98%) was purchased from Must (Chengdu, China) BIOTECHNOLOGY Co., Ltd. (Chengdu, China). Chromatographic methanol and acetonitrile were purchased from Merck KGaA (Darmstadt, Germany). Ethyl carbamate was purchased from Sinopharm Chemical Reagent Co., Ltd. (LOT20221021, Shanghai, China). The kits for sodium, potassium and chloride ions were purchased from Nanjing Jiancheng Bioengineering Institute (LOT20221013, Nanjing, China). The ultrapure water was prepared by the Milli-Q purification system (Darmstadt, Germany).

### 2.2. Animal Experiments

Male SD rats (180–220 g) were purchased from Changsha Tianqin Biotechnology Co., Ltd. (Changsha, China) and raised in the experimental animal center with the condition of 24 °C and RH 50%. Animal experiments were carried out in accordance with the Guiding Opinions of the Animal Ethics Committee of Guizhou University of Traditional Chinese Medicine. Male SD rats were selected for the diuretic study to avoid potential confounding effects of the estrous cycle on renal function and fluid-electrolyte balance, thereby minimizing inter-individual variability and ensuring the reliability of pharmacodynamic data.

Rats were randomly divided into five groups (*n* = 6 per group): control group (C), model group (M), positive control group (hydrochlorothiazide, 28 mg/kg), and two barbatic acid (BA) treatment groups (28 mg/kg and 56 mg/kg). The sample size of six rats per group was determined based on a combination of approaches: (1) Resource equation method: According to the degrees of freedom (E) approach for animal studies, E should fall between 10 and 20 for adequate statistical power without excessive animal use [15,16]. With six animals per group and five groups, the degrees of freedom for error (E) were calculated as E = total number of animals − number of groups = 30 − 5 = 25, which falls within the recommended range of 10–20. (2) Preliminary data: Based on our preliminary experiments, the observed effect size for urine output between BA-treated and model groups was approximately 1.5-fold with a coefficient of variation of ~20%, indicating that *n* = 6 would provide ≥ 80% power to detect significant differences at α = 0.05 (two-tailed). (3) Literature precedent: This sample size is consistent with previous diuretic studies in rat models and adheres to the 3R principle (Replacement, Reduction, Refinement) by using the minimum number of animals required to obtain scientifically valid results. This approach also aligns with the latest NIH guidelines requiring transparent reporting of sample size justification in animal studies [17].

The BA doses were selected based on preliminary experiments in which lower doses (7 and 14 mg/kg) showed no significant diuretic effects; therefore, the dose was doubled from 14 mg/kg to obtain 28 and 56 mg/kg for formal evaluation. The hydrochlorothiazide dose (28 mg/kg) was chosen based on preliminary tests showing that 10 and 20 mg/kg failed to significantly increase urinary electrolyte excretion, whereas 28 mg/kg (within the reported effective range [18]) produced significant effects.

A water-loaded model was established by intragastric administration of 0.9% sodium chloride solution (2.5 mL/100 g body weight). Following administration, the bladder was gently compressed to ensure complete voiding, and rats were immediately placed in metabolic cages for urine collection. Urine samples were collected hourly over a 6 h period.

The urine samples obtained within 6 h were used to detect the levels of Na^+^, K^+^ and Cl^−^ according to the method of kits. The urine and serum collected within 24 h were used for metabolomics identification. The kidney tissues were used for HE staining.

### 2.3. Metabonomics Research

#### 2.3.1. Sample Pretreatment

Mixing 100 μL samples and 400 μL 80% methanol aqueous solution evenly obtained a mixed solution placed in an ice bath for 5 min and centrifuged at 15,000× *g* at 4 °C for 20 min to precipitate proteins. Then the supernatant was diluted with pure water until the methanol content was 53%. The diluted samples were centrifuged at 15,000× *g* at 4 °C for 20 min for LC-MS/MS analysis.

In addition, the QC samples were prepared by mixing equal volumes of each experimental sample. The sample processing is the same as the above operation.

#### 2.3.2. LC-MS/MS Conditions

Metabolite analysis was performed on the AB SCIEX Triple TOF 6600 by Gene Denovo Biotechnology Co., Ltd. (Guangzhou, China). The scanning range was selected as *m*/*z* 100–1500. The settings for the ESI source were as follows: Spray voltage: 3.2 kV; Sheath gas flow rate: 40 arb; Auxiliary gas flow rate: 10 arb; Capillary temperature: 320 °C; Polarity: Positive and negative mode; the secondary scanning: MS/MS.

Both positive and negative electrospray ionization (ESI^+^ and ESI^−^) modes were employed to maximize metabolite coverage, as different classes of metabolites exhibit preferential ionization in one mode over the other [19,20]. Basic compounds (e.g., amines) ionize better in positive mode, while acidic compounds (e.g., carboxylic acids, phenolic acids) form stable [M − H]^−^ ions in negative mode [21]. This complementary approach ensures comprehensive profiling of the urinary and serum metabolomes.

The chromatographic column was Hypesil Gold column C18 (100 × 2.1 mm, 1.9 μm), the column temperature retained 40 °C, and the flow rate was 0.2 mL/min. The injection volume was 2 μL. In the positive ion mode, mobile phase A was 0.1% formic acid in water, and mobile phase B was methanol. In the negative ion mode, mobile phase A was 5 mM ammonium acetate with a pH of 9.0, and mobile phase B was methanol. The solvent gradient was set as follows: 2% B for 1.5 min; 100% B for 12.0 min; 100% B for 14.0 min; 2% B for 14.1 min; 2% B for 17 min. The chromatographic conditions were adapted from previously described metabolomics methods for biofluid analysis using reversed-phase LC-MS [22], with minor modifications to optimize separation for urine and serum samples.

The stability of the LC-MS system was assessed by injecting quality control (QC) samples at regular intervals throughout the analytical run. The retention time RSDs of the ten randomly selected metabolites in QC samples were all below 5%, indicating excellent chromatographic stability.

#### 2.3.3. Untargeted Metabolite Identification

The raw data files were imported into the CD 3.1 software to screen retention time and mass-to-charge ratio. Then, peak alignment was performed according to the retention time deviation of 0.2 min and the mass deviation of 5 ppm. Subsequently, peak extraction was carried out by parameters: the mass deviation of 5 ppm, the signal intensity deviation of 30%, the signal-to-noise ratio of 3, the minimum signal intensity of 100,000, and the peak areas were quantified. Next, the molecular formula predicted through molecular ion peaks and fragment ions were compared with the mzCloud (https://www.mzcloud.org/, accessed on 19 March 2026), mzVault and Masslist databases. Finally, the identification and quantitative results of the data were obtained.

Metabolomic profiling was performed in collaboration with Gene Denovo Biotechnology Co., Ltd. (Guangzhou, China) including mass spectrometry analyses.

### 2.4. Pharmacokinetic Study

A schematic overview of the pharmacokinetic study workflow is provided in Figure S1.

#### 2.4.1. Sample Preparation

Male SD rats (180–220 g) were purchased from Huafukang Biotechnology Co., Ltd. (Beijing, China) and the raising environment was the same as Section 2.3.1. Male rats were chosen for the pharmacokinetic study to eliminate the influence of the estrous cycle on drug-metabolizing enzyme activities, thus providing more consistent and interpretable pharmacokinetic parameters.

Male SD rats were randomly divided into three groups (*n* = 6 per group) receiving BA at 28, 56, or 112 mg/kg by intragastric administration (10 mL/kg). The oral gavage volume of 10 mL/kg was selected based on established guidelines for rodent dosing, which recommend a maximum volume of 10–20 mL/kg for rats to ensure safe and well-tolerated administration [23,24]. This volume is widely used in pharmacokinetic and toxicological studies [25,26,27] and is considered standard for aqueous or dissolved test compounds. The 10 mL/kg volume ensured consistent delivery of BA across all dose groups (28, 56, and 112 mg/kg) without exceeding gastric capacity or causing undue stress to the animals.

The 112 mg/kg dose was included to explore BA disposition at a supra-pharmacological level; it was not used in the efficacy study because pharmacokinetic analysis revealed nonlinear behavior at this dose, characterized by decreased volume of distribution and shortened half-life (see Section 3.3), precluding reliable extrapolation of dose–response relationships. All rats were fasted but allowed access to water for 12 h before BA administration.

**Figure 1:**
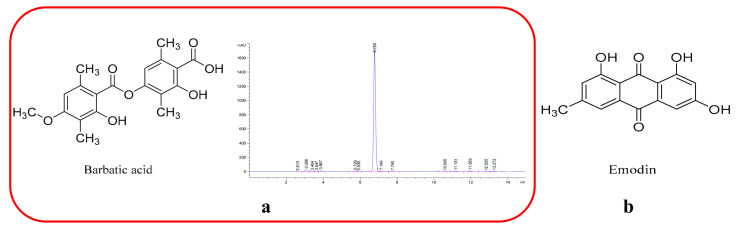
Chemical structures of barbatic acid and emodin. (**a**) Chemical structure of barbatic acid (BA) with purity diagrams; (**b**) chemical structure of emodin (internal standard).

As shown in Figure 1, Emodin was selected as the internal standard (IS) based on its structural similarity to barbatic acid (both are phenolic compounds with anthraquinone-like structures), which ensures comparable extraction recovery and chromatographic behavior during sample preparation and analysis [28,29]. Emodin exhibits excellent stability in rat plasma and does not interfere with the detection of BA under the established chromatographic conditions. Both BA and emodin were detected in negative electrospray ionization (ESI^−^) mode, as phenolic compounds readily lose protons to form stable [M − H]^−^ ions, providing superior sensitivity and signal-to-noise ratios compared to positive mode [30]. The MRM transitions monitored were *m*/*z* 359.2 → 177.1 for BA and *m*/*z* 269.0 → 225.1 for emodin, with collision energies optimized at –45 eV and –35 eV, respectively.

Blood samples were collected at 0, 0.033, 0.083, 0.17, 0.33, 0.5, 0.67, 1, 2, 4, 6, 8, 10, 12, 24, 48, and 72 h after administration into 1.5 mL tubes containing heparin sodium. Plasma was obtained by centrifugation at 3000 rpm for 15 min at 4 °C. The sampling schedule was designed based on preliminary data to ensure adequate characterization of the absorption, distribution, and elimination phases, with the 72 h time point capturing nearly the entire drug exposure and enabling reliable estimation of elimination kinetics.

10 μL plasma was added 190 μL methanol containing IS (10 ng/mL emodin) to precipitate proteins. Then the mixed solution was vortexed for 3 min and centrifuged at 3000 r/min for 10 min at 4 °C. The supernatant was transferred to another new EP tube, and dried with nitrogen at 37 °C, and added 200 μL methanol to redissolve it, then centrifuged for 5 min. Next, the supernatant was taken into the liner tube for detection.

#### 2.4.2. Instrument Conditions

Pharmacokinetic studies were conducted by the U3000-Q Exactive Focus UPLC system (Thermo Fisher Scientific, Dreieich, Germany) combination with the AB SCIEX Triple Quad™ 5500+ system (AB Sciex, Framingham, MA, USA) equipped with an electrospray ionization (ESI) source. The parameters of the negative ion mode were set as follows: Nitrogen was used as the nebulizer and auxiliary gas. The flow rates of the nebulizer gas (Gas 1), the heater gas (Gas 2) and the curtain gas were 55, 55 and 35 psi, respectively. The turbo spray temperature was 550 °C. The ion spray voltage was floated at −4500 V, and the declustering potential was −100 V. The collision energy (CE) was −45 eV, and the CE spread was set at 15 eV. The range of TOF scanning was set from *m*/*z* 100 to *m*/*z* 1200.

The UPLC was equipped with the ACQUITY UPLCR HSS T3 column (Waters, Milford, MA, USA, 1.8 μm 2.1 × 100 mm). Mobile phase was 0.1% formic acid-aqueous solution (A) and acetonitrile (B). The Gradient elution was 60% B at 2 min, 100% B at 8 min, 100% B at 10 min, and 60% B at 13 min. The flow rate was 0.3 mL/min, the column temperature was 35 °C, the injection volume was 2 μL. The UPLC-MS/MS method was developed based on published procedures for quantification of phenolic acids in rat plasma [31], with modifications to optimize sensitivity for BA and the internal standard (emodin).

#### 2.4.3. Methodology Investigation

The lower limit of quantification (LLOQ) was 0.125 ng/mL for BBS. The good linear range was 0.125~2500 ng/mL for BBS. The intra-day and inter-day precisions were both less than 15%. The average recovery rate of BBS was between 93% and 97%, and the matrix effect was between 90% and 95%. Detailed methodology validation data, including precision, accuracy, recovery, matrix effect, and stability, are provided in Supplementary Tables S1–S4. Representative chromatograms of blank plasma, spiked plasma, and dosed plasma samples are provided in Supplementary Figures S2–S4, respectively.

### 2.5. Statistical Analysis

#### 2.5.1. Pharmacological Active Analysis

All data were expressed as mean ± standard deviation. Statistical analysis was performed by SPSS 26.0 software. Comparisons between treatment groups and the model group were performed using one-way ANOVA followed by Dunnett’s post hoc test. Given the a priori hypothesis that BA would increase urinary electrolyte excretion, one-tailed tests were applied for these comparisons, and *p*-values < 0.05 were considered statistically significant.

#### 2.5.2. Metabonomics Analysis

The data were normalized through log10 transformation before statistical analysis. Multivariate statistics and pathway analysis were carried out using MetaboAnalyst 3.0 (www.metaboanalyst.ca, accessed on 19 March 2026), including principal component analysis (PCA), partial least squares discriminant analysis (PLS-DA), and orthogonal partial least squares discriminant analysis (OPLS-DA). Based on the variable importance of (VIP) > 1 and the *p*-value (*t*-test) < 0.05, differential metabolites were selected from KEGG pathways for enrichment analysis.

#### 2.5.3. Pharmacokinetic Analysis

The pharmacokinetic parameters were calculated by the DAS 2.0 pharmacokinetic software, including AUC_0–t_, AUC_0–∞_, t_1/2_, Tmax, CLz/F, Vz/F, and Cmax.

## 3. Results

### 3.1. Pharmacodynamic Evaluation

#### 3.1.1. The Urine Volume Changes

**Figure 2:**
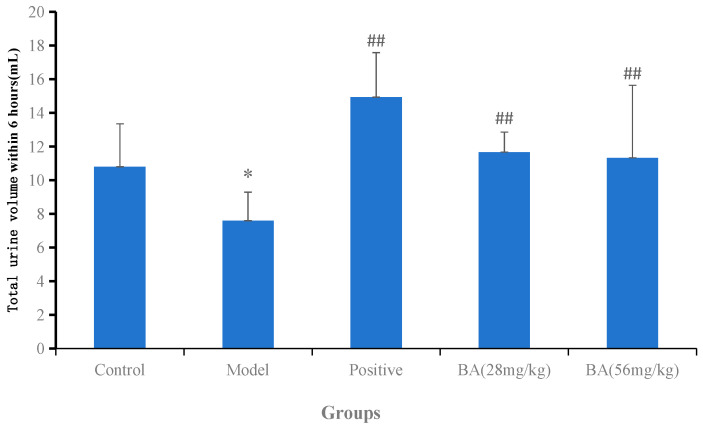
Total urine volume within 6 h after BA treatment. Data are presented as mean ± SD (*n* = 6 per group). Statistical analysis was performed using one-way ANOVA followed by Dunnett’s post hoc test for multiple comparisons. Compared with control group: * *p* < 0.05. Compared with model group: ## *p* < 0.01.

**Figure 3:**
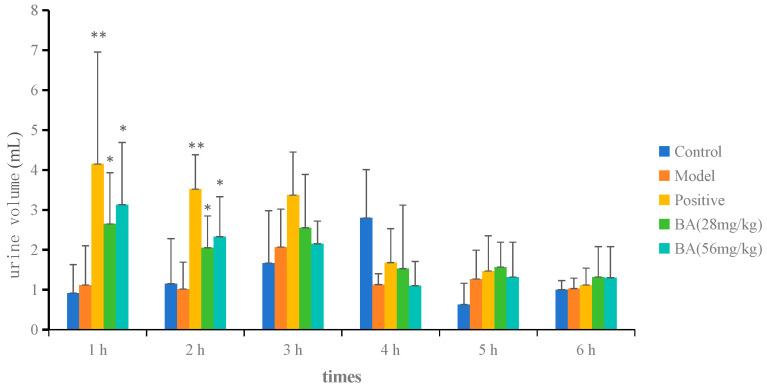
Urine volume at each time point after BA treatment. Data are presented as mean ± SD (*n* = 6 per group). Statistical analysis was performed using one-way ANOVA followed by Dunnett’s post hoc test for multiple comparisons. Compared with control group: * *p* < 0.05, ** *p* < 0.01.

As shown in Figure 2 and Table S5, the total urine volume collected over 6 h in the model group (7.60 ± 1.70 mL) was significantly lower than that in the control group (10.80 ± 2.55 mL). Following treatment with BA (28 and 56 mg/kg), the total urine volume increased markedly to 11.67 ± 1.19 mL and 12.05 ± 1.32 mL, respectively (*p* < 0.01 vs. model), indicating a significant diuretic effect. The time course of urine excretion (Figure 3 and Table S6) revealed that BA significantly enhanced urine output during the first 2 h, after which the effect gradually diminished from 3 to 6 h. These results indicate that BA exerts a rapid but short-lasting diuretic effect, with the most pronounced increase occurring within the first 2 h post-administration.

#### 3.1.2. Detection the Levels of Na^+^, K^+^ and Cl^−^

**Figure 4:**
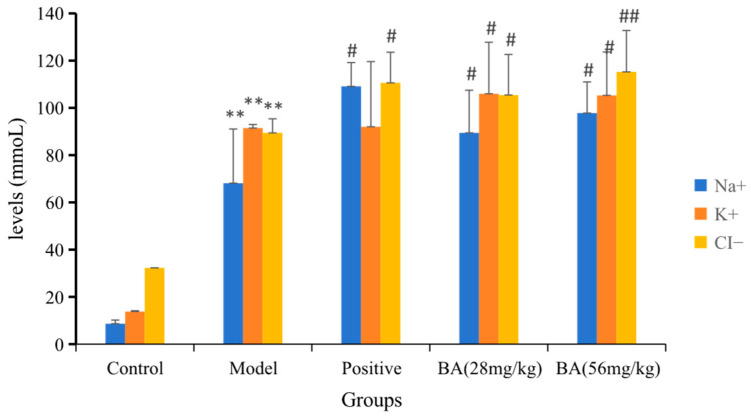
The Effect on the level of Na^+^, K^+^ and Cl^−^ in urine of water-loaded rats within 6 h after BA treatment. Data are presented as mean ± SD (*n* = 6 per group). Statistical analysis was performed using one-way ANOVA followed by Dunnett’s post hoc test for multiple comparisons. Compared with control group: ** *p* < 0.01. Compared with model group: # *p* < 0.05; ## *p* < 0.01.

As shown in Figure 4 and Table S7, the urinary excretion levels of Na^+^, K^+^, and Cl^−^ were significantly elevated in the model group compared with the control group, confirming successful water-loading. Following BA treatment, the levels of Na^+^, K^+^, and Cl^−^ were further increased, with the increase in Cl^−^ excretion being statistically more significant. Specifically, BA at 28 and 56 mg/kg increased Na^+^ excretion by 1.3- and 1.4-fold, K^+^ excretion by 1.2 fold, and Cl^−^ excretion by 1.2- and 1.3-fold, respectively (*p* < 0.05–0.01 vs. model). Detailed effect sizes and confidence intervals for all comparisons are provided in Supplementary Table S8. These findings demonstrate that BA promotes natriuresis and chloriuresis accompanied by kaliuresis, a pattern most closely resembling that of thiazide or loop diuretics.

#### 3.1.3. Results of Haematoxylin and Eosin

**Figure 5:**
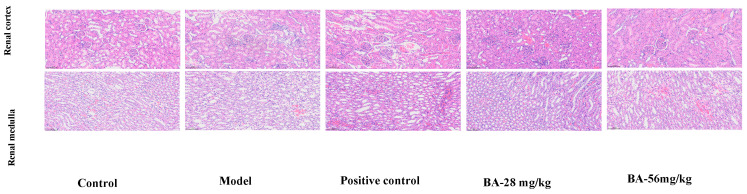
Staining diagram of renal pathological HE in rats in each group (All images are shown at 200× magnification; scale bar = 100 μm).

As shown in Figure 5, in the renal cortex area, the structures of the collecting ducts and glomeruli in all experimental groups were clear and intact, and no damage was found. The epithelial cells of the collecting ducts were neatly arranged without the phenomena of detachment or swelling. No abnormal dilation was found in the capillary lumen or the renal capsule cavity, and the renal tubular cells did not show obvious pathological changes in the Group M and the BA treatment. In the renal medulla area, the morphology of the collecting ducts was clearly visible. The dilation of the collecting ducts was obvious in the BA and positive drug treatment. These observations confirm that BA does not induce cortical damage, and the medullary dilation is consistent with its diuretic action.

### 3.2. Metabonomics Results

#### 3.2.1. Multivariate Statistical Analysis

Urinary metabolomics identified 542 and 249 metabolites in positive ion (POS) and negative ion (NEG) modes, respectively. Quality control (QC) samples clustered tightly together in the PCA score plot, indicating good instrument stability and data reproducibility. A clear separation among all groups was observed in the OPLS-DA score plot (Figure S5A). Cross-validation of the urinary OPLS-DA model yielded R^2^Y = 0.984 and Q^2^ = 0.825, demonstrating excellent goodness-of-fit and predictive ability. Similarly, blood metabolomics analysis identified 641 and 705 metabolites in POS and NEG modes, respectively, with complete group separation achieved in the OPLS-DA score plot (Figure S5B). Model validation using 200 permutation tests confirmed robustness (R^2^Y = 0.987, Q^2^ = 0.833, *p* < 0.05). These results confirm that BA treatment induces distinct metabolic alterations in both urine and serum.

#### 3.2.2. Identification of Differential Metabolites

Differential metabolites were selected based on VIP > 1 and *p* < 0.05. In urine, 75 metabolites differed between control and model groups, and 34 metabolites differed between model and BA-treated groups (56 mg/kg). In serum, 29 metabolites differed between control and model, and 34 metabolites differed between model and BA-treated. The up-regulated and down-regulated metabolites across comparisons are shown in Figure S6 and volcano plots (Figure S7). Common differential metabolites between the C-M and M-BA comparisons included 14 in urine (Table 1) and 2 in serum (Table 2). Notably, S-adenosylmethionine, uric acid, and 3-oxoindane-1-carboxylic acid were up-regulated in the model group and reversed by BA. These data reveal that BA modulates a distinct set of metabolites, many of which are involved in amino acid metabolism.

Complete lists of differential metabolites identified in urine and serum samples, including fold-change directions, *p*-values, and VIP scores, are provided in Supplementary Tables S9 and S11 (C vs. M) and Tables S10 and S12 (M vs. BA). A small number of differential metabolites, such as the feature labeled ‘FLK’, could not be identified through database matching and are therefore presented with only their accurate mass and retention time. Their inclusion reflects their statistical contribution to group separation, but their biological relevance remains to be elucidated.

#### 3.2.3. KEGG Functional Enrichment Analysis

To identify the biological pathways affected by BA, KEGG enrichment analysis was performed on the differential metabolites. In urine, BA primarily modulated pathways including the citrate cycle (TCA cycle), riboflavin metabolism, nicotinate and nicotinamide metabolism, and multiple amino acid metabolism pathways (cysteine and methionine, alanine/aspartate/glutamate, arginine/proline, glycine/serine/threonine, tyrosine, histidine, and taurine/hypotaurine metabolism) (Table 3, Figure S8). In serum, BA affected primary bile acid biosynthesis, phenylalanine metabolism, taurine/hypotaurine metabolism, valine/leucine/isoleucine biosynthesis, vitamin B6 metabolism, and arginine/proline metabolism (Table 4, Figure S9). Topological analysis further confirmed the importance of these pathways (Figure S9). Collectively, these findings indicate that BA’s diuretic mechanism is strongly associated with the regulation of amino acid metabolism and related pathways.

### 3.3. Pharmacokinetic Results

**Figure 6:**
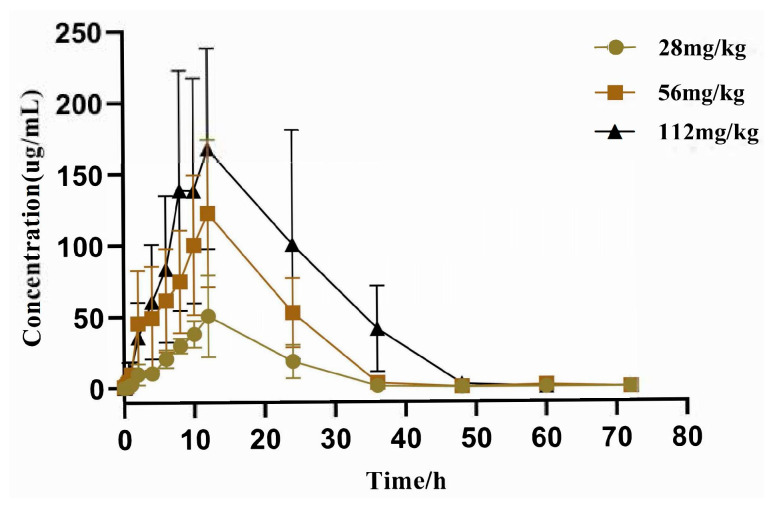
The pharmacokinetic profiles of BA in rats after the oral administration of 28 mg/kg, and 56 mg/kg and 112 mg/kg). Each point represents the mean ± SD of six determinations.

To characterize the in vivo behavior of BA, a pharmacokinetic study was conducted in rats following oral administration at 28, 56, and 112 mg/kg. The mean plasma concentration-time curves are shown in Figure 6, and the main pharmacokinetic parameters are summarized in Table 5.

BA exhibited dose-dependent increases in systemic exposure. The AUC_0–t_ increased from 581.73 ± 7.54 μg·h·L^−1^ at 28 mg/kg to 4775.96 ± 92.62 μg·h·L^−1^ at 112 mg/kg, and Cmax rose from 36.35 ± 1.47 μg/L to 224.80 ± 21.87 μg/L. The low coefficients of variation (CV%) for AUC_0–t_ (0.70–1.94%) and Cmax (4.04–9.73%) indicate excellent reproducibility and low inter-individual variability for these core exposure metrics.

The terminal half-life (t_1/2_) decreased with increasing dose, from 5.88 ± 0.64 h at 28 mg/kg to 2.61 ± 0.99 h at 112 mg/kg, accompanied by a marked reduction in the apparent volume of distribution (Vz/F: from 0.27 ± 0.20 L/kg to 0.088 ± 0.03 L/kg). These changes suggest saturation of tissue distribution or elimination processes at the highest dose. Clearance (CLz/F) remained low across doses (0.023–0.034 L·h·kg^−1^), indicating slow elimination. Moderate to high variability was observed for t_1/2_ (CV%: 10.9–44.6%), Vz/F (34.1–74.1%), and CLz/F (38.5–58.8%), reflecting inter-individual differences in drug distribution and elimination. The time to peak concentration (tmax) ranged from 10.67 to 12.00 h across doses, with minimal variability (CV% ≤ 10.8%).

For all dose groups, the ratio of AUC_0–t_ to AUC_0–∞_ exceeded 99.8%, confirming that the 72 h sampling period captured nearly complete drug exposure and was adequate for characterizing the elimination phase. These results demonstrate that BA has favorable exposure at active doses (28–56 mg/kg) but exhibits nonlinear disposition at the suprapharmacological dose of 112 mg/kg, supporting the exclusion of this dose from efficacy studies and providing a basis for subsequent pharmacodynamic analyses.

## 4. Discussion

In this study, BA administration significantly increased 6 h urine output in water-loaded rats, with the most pronounced effect occurring within the first 2 h (Figure 2 and Figure 3). This rapid onset of action suggests that BA or its active metabolites may reach the renal tubules quickly following oral absorption, while the short duration implies either rapid elimination or transient target engagement. The pharmacokinetic profile of BA (t_1/2_ ~5–6 h at active doses) supports this interpretation, as the diuretic effect subsided within 3–4 h despite measurable plasma concentrations, suggesting that the pharmacodynamic effect may not directly mirror plasma exposure—a phenomenon often observed with drugs that act via receptor binding or intracellular signaling rather than direct tubular interactions. It should be noted that baseline urine output was not recorded in this study because the bladders were manually voided immediately after saline loading to standardize the starting point for urine collection. While this procedure ensures consistency in the initial bladder volume, the absence of baseline data limits our ability to calculate the net diuretic effect relative to pre-treatment levels. Future studies should include a baseline collection period to enable more precise quantification of drug-induced changes.

To contextualize BA’s mechanism, it is useful to compare its effects with those of established diuretic classes [32]. Loop diuretics (e.g., furosemide) inhibit the Na^+^-K^+^-2Cl^−^ cotransporter (NKCC2) in the thick ascending limb, producing potent but short-lasting diuresis with significant Na^+^ and Cl^−^ excretion [33,34]. Thiazide diuretics target the Na^+^-Cl^−^ symporter (NCC) in the distal convoluted tubule, resulting in moderate diuresis and are first-line agents for hypertension [34,35,36]. Carbonic anhydrase inhibitors (CAIs) act in the proximal tubule but are rarely used as primary diuretics due to limited efficacy and metabolic acidosis risk [32]. Potassium-sparing diuretics (e.g., amiloride) block epithelial sodium channels (ENaC) in the collecting duct, minimizing K^+^ loss but producing weak diuresis. Osmotic diuretics (e.g., mannitol) act by increasing tubular fluid osmolality, with indications limited to specific clinical settings [32].

BA increased urinary excretion of Na^+^, K^+^, and Cl^−^ (Figure 4), a pattern most closely resembling that of thiazide or loop diuretics, both of which promote natriuresis and chloriuresis accompanied by variable K^+^ loss. The significant kaliuresis observed with BA suggests that, like thiazides and loop diuretics, it may inhibit sodium reabsorption proximal to the K^+^-secreting sites in the distal nephron, thereby increasing distal Na^+^ delivery and enhancing K^+^ secretion. This raises a clinically relevant concern: prolonged BA use could potentially induce hypokalemia, a well-known adverse effect of thiazide and loop diuretics. However, unlike loop diuretics, BA did not produce a rapid, high-ceiling diuresis; its effect size (~1.5-fold increase over model) is more consistent with thiazide-like moderate diuresis. Notably, BA’s action appears distinct from potassium-sparing diuretics (which reduce K^+^ excretion) and CAIs (which primarily affect HCO_3_^−^ excretion), suggesting a mechanism involving inhibition of sodium reabsorption in the distal convoluted tubule or connecting segment.

Beyond the pharmacodynamic comparison, a metabolomic perspective provides further insights into BA’s distinct profile relative to standard diuretics. While the present study did not include a direct metabolomic comparison with classical diuretics under identical conditions, published data indicate that thiazide diuretics are associated with metabolic disturbances such as hyperuricemia and dyslipidemia [37]. In contrast, BA treatment was associated with reduced urinary uric acid excretion and no significant alterations in lipid metabolism, suggesting a potentially favorable metabolic safety profile. Furthermore, the prominent modulation of multiple amino acid metabolism pathways observed with BA has not been prominently reported for classical diuretics, although recent evidence suggests that loop diuretics may influence specific amino acid pathways such as tryptophan metabolism [38]. These observations highlight the need for future head-to-head metabolomic studies comparing BA with standard diuretics (e.g., hydrochlorothiazide, furosemide) under identical experimental conditions to definitively characterize the similarities and differences in their metabolic fingerprints.

An intriguing finding was the differential effect of BA on uric acid metabolism compared to classical diuretics. Thiazide diuretics are known to increase serum uric acid by enhancing proximal tubular reabsorption, contributing to hyperuricemia and gout risk in susceptible patients [39]. In contrast, BA treatment significantly reduced urinary uric acid excretion in the water-loaded model (metabolomics data), suggesting it may lower, rather than elevate, uric acid levels. This could represent a clinically meaningful advantage if confirmed in future studies. Metabolomic profiling revealed that BA treatment is associated with alterations in multiple amino acid metabolism pathways, including cysteine and methionine metabolism, tyrosine metabolism, histidine metabolism, taurine hypotaurine metabolismand, and phenylalanine metabolism (Table 3 and Table 4). Beyond their role as metabolic intermediates, several of these amino acids have direct physiological functions in the kidney. For instance, taurine, glycine, and proline are known to act as organic osmolytes that accumulate in renal medullary cells to counteract the high extracellular osmolarity, thereby protecting cells from osmotic stress and maintaining cell volume during urine concentration [40]. BA-induced alterations in these amino acids could therefore influence the kidney’s ability to handle osmotic gradients, potentially affecting water reabsorption and urine concentrating capacity. Additionally, amino acid metabolism is closely linked to acid-base balance: glutamine (a key metabolite in the arginine and proline metabolism pathway) is a major substrate for renal ammoniagenesis, contributing to acid-base homeostasis [41]. In response to acidosis, the kidney increases glutamine uptake and metabolism to produce ammonium (NH_4_^+^) for urinary acid excretion. While our study did not directly measure ammonium or blood pH, the observed changes in glutamine-related pathways suggest that BA may modulate renal acid excretion, which could have implications for systemic acid-base status during prolonged diuretic therapy.

These findings suggest that the diuretic effect of BA may involve modulation of amino acid metabolism. It is well recognized that amino acid metabolites serve as precursors for purine synthesis, and purines are substrates for uric acid production [42]. Based on these observations, it is reasonable to hypothesize that BA may reduce urinary uric acid through regulation of amino acid metabolism, potentially contributing to its diuretic action. However, it is important to note that these findings represent pathway inferences derived from untargeted metabolomics rather than direct molecular evidence. Further experimental studies—such as targeted enzyme assays, gene expression analysis, or investigation of renal transporter activity (e.g., NKCC2, NCC)—are needed to establish causal relationships and confirm the proposed mechanisms underlying BA’s diuretic effect. Furthermore, no significant lipid metabolism disturbances were observed in BA-treated rats, contrasting with the well-documented dyslipidemic effects of thiazides [43]. If confirmed, this could position BA as a diuretic with a favorable metabolic safety profile, though targeted lipidomic studies are needed to substantiate this preliminary observation.

It should also be noted that a small number of differential metabolites, such as the feature designated as “FLK” in Table 1 and Table 2, could not be identified through database matching (mzCloud, mzVault, or MassList). These metabolites are reported based on their accurate mass and retention time, and their inclusion reflects their statistical contribution to group separation (VIP > 1, *p* < 0.05). However, their biological interpretation is currently limited and awaits future structural elucidation and functional studies.

Furthermore, the key metabolites identified in this study—particularly those involved in amino acid metabolism pathways (e.g., taurine, glutamine, glycine)—should be validated using targeted LC-MS/MS with authentic standards to confirm their differential abundance and enable absolute quantification. Such validation would strengthen the reliability of our metabolomic findings. More importantly, future studies should investigate the correlation between these validated metabolites and established renal function markers (e.g., glomerular filtration rate, urinary neutrophil gelatinase-associated lipocalin [NGAL], kidney injury molecule-1 [KIM-1], or β2-microglobulin). Establishing such correlations would help elucidate whether the observed metabolic changes are directly linked to BA’s diuretic effect and renal protection, rather than being epiphenomena.

Beyond electrolyte handling, BA may influence renal function through metabolic effects that ultimately impact key ion transporters. The observed modulation of riboflavin metabolism is noteworthy, as riboflavin (vitamin B_2_) is a precursor for flavin coenzymes (FAD, FMN) essential for mitochondrial energy production [44]. By potentially enhancing riboflavin availability or utilization, BA could support the high energy demands of renal tubular transport, particularly active Na^+^ reabsorption mediated by Na^+^/K^+^-ATPase, thereby indirectly contributing to diuretic efficiency. Furthermore, the energy-dependent activity of apical transporters such as NCC (thiazide-sensitive Na^+^-Cl^−^ symporter) and NKCC2 (loop diuretic-sensitive Na^+^-K^+^-2Cl^−^ cotransporter) could be indirectly influenced by changes in cellular energy status. In addition, alterations in amino acid metabolism may affect the expression or function of aquaporins (AQPs), particularly AQP2 in the collecting duct, through changes in medullary osmolyte concentration or urea cycling [45]. However, these linkages remain speculative, as our study did not directly measure transporter expression, localization, or activity. Future studies should investigate whether BA modulates the expression of NCC, NKCC2, AQP2, or Na^+^/K^+^-ATPase using Western blot, immunohistochemistry, or qPCR, and assess transporter function through electrophysiological or isolated tubule perfusion experiments.

Integrating the pharmacokinetic and pharmacodynamic data provides further mechanistic insight. BA exhibited dose-dependent pharmacokinetics, with half-lives of 5.88 and 5.23 h at 28 and 56 mg/kg, respectively, but a shortened t_1/2_ (2.61 h) and reduced Vz/F at 112 mg/kg (Table 5). This nonlinear disposition at the highest dose—characterized by decreased volume of distribution and accelerated elimination—suggests saturation of tissue binding or altered protein binding at suprapharmacological concentrations. In addition to saturation of tissue binding, the nonlinear behavior may also reflect saturation of metabolic or elimination pathways. BA is a phenolic compound that is likely metabolized by phase II enzymes (e.g., UDP-glucuronosyltransferases, sulfotransferases) in the liver and kidney [46]. At high concentrations, these enzymes may become saturated, leading to a disproportionate increase in parent drug exposure and a shift in elimination kinetics. Alternatively, saturation of efflux transporters (e.g., P-glycoprotein, MRP2) involved in biliary or renal excretion could contribute to the observed changes in half-life and clearance [47,48]. The accelerated elimination (shortened t_1/2_) despite reduced Vz/F suggests that the net effect may involve complex interplay between distribution and elimination processes, possibly with different saturable components. Importantly, this nonlinearity precluded reliable extrapolation of efficacy from lower doses, justifying the exclusion of 112 mg/kg from the pharmacodynamic study. The concordance between systemic exposure (AUC) and diuretic effect at 28–56 mg/kg supports the relevance of this dose range for future translational studies.

While the concordance between AUC and diuretic effect at 28 and 56 mg/kg suggests a positive exposure–response relationship within the therapeutic range, a formal quantitative analysis (e.g., correlation or regression) was not performed due to the limited number of doses (*n* = 2) in the linear range. The consistent increase in both AUC and urine output from 28 to 56 mg/kg nonetheless supports that BA’s diuretic action is exposure-dependent. Future studies incorporating a broader dose range and individual-level PK/PD modeling (e.g., Emax models) are warranted to precisely characterize the exposure–response relationship and guide dose selection for clinical development.

Notably, the Tmax (10–12 h) lagged behind the peak diuretic effect (within 2 h). This relatively long Tmax suggests delayed absorption, which may be attributed to several factors. First, BA is a lipophilic phenolic compound, and highly lipophilic drugs often undergo incorporation into chylomicrons in the intestine, leading to lymphatic transport rather than direct portal venous absorption [49]. This lymphatic pathway can result in a delayed and prolonged absorption phase. Second, the aqueous solubility of BA may be limited, and slow dissolution in the gastrointestinal tract could extend the time required to reach peak plasma concentrations [50]. Third, the possibility of enterohepatic circulation cannot be excluded, which might contribute to a secondary absorption peak and an apparently prolonged Tmax [51]. While these explanations are plausible, the exact mechanism underlying BA’s delayed absorption remains to be elucidated. Future studies using different formulations (e.g., solubilized formulations, nanoparticles) or administration routes (e.g., intraduodenal vs. oral) could help clarify whether the delay is due to physicochemical properties or physiological factors such as gastric emptying or intestinal transit time.

The dissociation between Tmax and peak diuretic effect indicates that BA’s diuretic action may be mediated by metabolites or triggered by an initial peak concentration that is not sustained. Alternatively, BA might exert its effect through receptor binding with rapid onset but short residence time. Future studies should explore whether BA or its metabolites directly interact with renal transporters using in vitro systems (e.g., oocyte expression assays or isolated perfused tubules) to definitively establish its molecular mechanism.

Regarding the histological assessment, it should be noted that the evaluation of tubular dilation in this study was based on qualitative description rather than quantitative scoring. While the observed dilation was clearly evident in BA-treated and positive control groups, the lack of a standardized semi-quantitative scoring system limits the objectivity and reproducibility of these findings. Future studies should implement a blinded, semi-quantitative scoring system (e.g., 0–3 scale based on the extent and severity of dilation) with independent assessment by multiple pathologists to enable statistical comparison and more rigorous interpretation of drug-induced renal effects.

Several limitations regarding the experimental design should also be acknowledged. The sample size of six rats per group, while statistically justified (Section 2.2), is relatively modest; larger cohorts would provide greater statistical power and enable more robust detection of smaller effect sizes. Additionally, the exclusive use of male rats, although chosen to avoid confounding effects of the estrous cycle, limits the generalizability of our findings to females. Furthermore, only a single rat strain (Sprague-Dawley) was employed; future studies should include both sexes and additional strains (e.g., Wistar rats) to confirm that the observed effects are strain-independent and to assess potential sex-specific differences in BA’s pharmacokinetics and pharmacodynamics.

## 5. Conclusions

This study provides the first integrated pharmacokinetic, pharmacodynamic, and metabolomic characterization of barbatic acid (BA) as a potential diuretic agent. In a water-loaded rat model, BA significantly increased urine output and promoted urinary excretion of Na^+^, K^+^, and Cl^−^, with an effect size and electrolyte profile most closely resembling that of thiazide diuretics. Unlike thiazides, however, BA treatment was associated with reduced urinary uric acid levels and no observable disturbance in lipid metabolism, suggesting a potentially more favorable metabolic safety profile. Metabolomic analysis revealed that BA modulates several amino acid metabolism pathways (glycine/serine/threonine, arginine/proline, tyrosine) and riboflavin metabolism, providing plausible, albeit hypothesis-generating, links to its diuretic mechanism. Pharmacokinetic studies showed dose-dependent behavior with moderate half-lives (5–6 h) at active doses (28–56 mg/kg) and nonlinear disposition at a suprapharmacological dose (112 mg/kg), which justified the exclusion of the highest dose from efficacy evaluation.

Nevertheless, several limitations of this study should be acknowledged. The pathway inferences derived from untargeted metabolomics are correlative and require direct experimental validation, such as targeted enzyme assays, transporter expression studies, or in vitro renal tubule models, to establish causality. The absence of direct molecular target identification (e.g., interaction with NKCC2, NCC, or renal transporters) and the lack of long-term safety assessment (e.g., effects on blood pressure, electrolyte balance, or bone metabolism) preclude definitive mechanistic conclusions. Future studies should investigate BA’s efficacy in disease-relevant models (e.g., hypertension, heart failure) and evaluate its translational potential through advanced preclinical toxicology and formulation development.

In summary, BA represents a promising natural-product-based diuretic candidate with a distinctive metabolic signature, warranting further mechanistic and translational investigation.

## Figures and Tables

**Table 1:** Differential metabolites involved in regulation in urine.

Number	Compounds	Formula	Rt/min	C-vs.-M	M-vs.-H	Mode
1	Isorhapontigenin	C_15_H_14_O_4_	5.155	↓	↓	POS
2	Riboflavin	C_17_H_20_N_4_O_6_	5.265	↓	↓	POS
3	Nicotinuric acid	C_8_H_8_N_2_O_3_	5.344	↓	↓	POS
4	S-Adenosylmethionine	C_15_H_22_N_6_O_5_S	2.038	↑	↓	POS
5	N-Acetylhistamine	C_7_H_11_N_3_O	1.327	↓	↓	POS
6	Uric acid	C_5_H_4_N_4_O_3_	1.421	↑	↓	NEG
7	cis-Aconitic acid	C_6_H_6_O_6_	1.37	↓	↑	NEG
8	2-Hydroxy-2-methylbutanedioic acid	C_5_H_8_O_5_	1.371	↓	↑	NEG
9	2-(acetyloxy)-3-amino-1-[1,2-di(acetyloxy)ethyl]-3-oxopropyl acetate	C_13_H_19_NO_9_	4.624	↓	↑	POS
10	N-[1-(4-methoxy-2-oxo-2H-pyran-6-yl)-2-methylbutyl] acetamide	C_13_H_19_NO_4_	5.149	↓	↓	POS
11	7-(2-aminophenyl) heptanoic acid	C_13_H_19_NO_2_	1.327	↓	↑	POS
12	FLK	C_21_H_34_N_4_O_4_	1.333	↓	↑	POS
13	3-oxoindane-1-carboxylic acid	C_10_H_8_O_3_	5.280	↑	↓	POS
14	3-methyl-5-oxo-5-(4-toluidino) pentanoic acid	C_13_H_17_NO_3_	1.285	↓	↑	POS

Note: ↑ indicates that the compound is upregulated; ↓ indicates that the compound has been downgraded. (FLK is an unidentified metabolite that was significantly altered (VIP > 1, *p* < 0.05) but could not be matched to any known compound in the databases. It is reported based on its accurate mass ([M + H]^+^ at *m*/*z* 204.1343, retention time 1.33 min) and molecular formula C_21_H_34_N_4_O_4_; its biological interpretation is currently limited and awaits future identification).

**Table 2:** Differential metabolites regulated by barbatic acid in blood.

Number	Compounds	Formula	Rt/min	C-vs.-M	M-vs.-H	Mode
1	2-Hydroxyvaleric acid	C_5_H_10_O_3_	5.271	↑	↓	NEG
2	Dodecanedioic acid	C_12_H_22_O_4_	6.335	↑	↓	NEG

Note: ↑ indicates that the compound is upregulated; ↓ indicates that the compound has been downgraded.

**Table 3:** The pathways of differential metabolites in urine.

Pathway Name	Match Status	*p*	−log10(p)	FDR	Impact
Citrate cycle (TCA cycle)	4/20	8.06 × 10^−5^	4.0936	0.00644	0.24395
Nicotinate and nicotinamide metabolism	2/15	0.014574	1.8364	0.29325	0.1943
Riboflavin metabolism	1/4	0.049881	1.3021	0.66508	0.5
Arginine and proline metabolism	2/36	0.074599	1.1273	0.74599	0.06628
Caffeine metabolism	1/10	0.12029	0.91976	0.96234	0
Arginine biosynthesis	1/14	0.16445	0.78397	1	0
Butanoate metabolism	1/15	0.17515	0.75658	1	0
Purine metabolism	2/70	0.22207	0.6535	1	0.01146
beta-Alanine metabolism	1/21	0.2367	0.6258	1	0
Lipoic acid metabolism	1/28	0.30299	0.51857	1	0
Glutathione metabolism	1/28	0.30299	0.51857	1	0.00719
Glycine, serine and threonine metabolism	1/33	0.34695	0.45973	1	0
Glycerophospholipid metabolism	1/36	0.37205	0.4294	1	0.02582
Tryptophan metabolism	1/41	0.41186	0.38525	1	0
Primary bile acid biosynthesis	1/46	0.44926	0.34751	1	0.00758
Cysteine and methionine metabolism	2/33	0.063949	1.1942	0.73508	0.15717
Glyoxylate and dicarboxylate metabolism	3/32	0.0068712	2.163	0.27485	0.05556
Alanine, aspartate and glutamate metabolism	2/28	0.047542	1.3229	0.66508	0.04808
Tyrosine metabolism	3/42	0.014663	1.8338	0.29325	0.10123
Taurine and hypotaurine metabolism	1/8	0.097392	1.0115	0.86571	0.42857
Histidine metabolism	1/16	0.18573	0.73112	1	0.12295

**Table 4:** The pathways of differential metabolites in serum.

Pathway Name	Match Status	*p*	−log10(p)	FDR	Impact
Primary bile acid biosynthesis	3/46	0.0011794	2.9283	0.094353	0.01563
Valine, leucine and isoleucine biosynthesis	1/8	0.040007	1.3979	0.71853	0
Phenylalanine metabolism	1/8	0.040007	1.3979	0.71853	0.14286
Taurine and hypotaurine metabolism	1/8	0.040007	1.3979	0.71853	0.42857
Glycine, serine and threonine metabolism	1/33	0.15615	0.80646	1	0
Tyrosine metabolism	1/42	0.19484	0.71032	1	0.00077
Steroid hormone biosynthesis	1/87	0.36595	0.43658	1	0.02538
Arginine and proline metabolism	2/36	0.0048663	2.3128	0.38931	0.04535
Biosynthesis of unsaturated fatty acids	1/36	0.10931	0.96133	1	0
Fatty acid degradation	1/39	0.11797	0.92823	1	0
Vitamin B6 metabolism	1/9	0.044908	1.3477	0.71853	0.07843

**Table 5:** Pharmacokinetic parameters for BA ($\bar{x}$ ± s, *n* = 6, with CV% in parentheses).

Parameters	28 mg/kg	56 mg/kg	112 mg/kg
AUC_0–t_ (μg·h·L^−1^)	581.73 ± 7.54 (1.30%)	2111.09 ± 14.70 (0.70%)	4775.96 ± 92.62 (1.94%)
AUC_0–∞_ (μg·h·L^−1^)	582.90 ± 7.60 (1.30%)	2111.57 ± 14.05 (0.67%)	4776.37 ± 93.09 (1.95%)
MRT (0–t)	13.18 ± 0.53 (4.02%)	15.95 ± 0.82 (5.14%)	18.25 ± 0.39 (2.14%)
MRT (0–∞)	13.35 ± 0.12 (0.90%)	15.95 ± 0.61 (3.82%)	18.26 ± 0.87 (4.76%)
t_1/2_ (h)	5.88 ± 0.64 (10.88%)	5.23 ± 2.33 (44.55%)	2.61 ± 0.99 (37.93%)
Tmax (h)	10.67 ± 1.15 (10.78%)	11.33 ± 0.32 (2.82%)	12 ± 0.01 (0.08%)
Vz/F (L/kg)	0.27 ± 0.20 (74.07%)	0.20 ± 0.09 (45.00%)	0.088 ± 0.03 (34.09%)
CLz/F (L·h·kg^−1^)	0.034 ± 0.02 (58.82%)	0.026 ± 0.01 (38.46%)	0.023 ± 0.01 (43.48%)
Cmax (μg/L)	36.35 ± 1.47 (4.04%)	96.36 ± 3.93 (4.08%)	224.80 ± 21.87 (9.73%)

## Data Availability

The original contributions presented in this study are included in the article. Further inquiries can be directed to the corresponding authors.

## Supplementary Materials

The following supporting information can be downloaded at: https://www.mdpi.com/article/10.3390/biology15070521/s1, Table S1: Precision and accuracy of the lower limit of quantitation for BA; Table S2: Precision and accuracy of BA in rat plasma; Table S3: Recovery Rate and Matrix Effect of Bate Acid in Plasma; Table S4: Stability of Barbatic acid in the rat plasma; Table S5: Total urine volume (mL) over 6 consecutive hours; Table S6: Urine volume (mL) at different time points after administration; Table S7: Effects of BA on urinary Na^+^, K^+^, and Cl^−^ excretion in water-loaded rats over 6 h; Table S8: Detailed effect sizes and confidence intervals for all comparisons; Table S9: Differential metabolites identified in urine samples of the C vs. M group; Table S10: Differential metabolites identified in urine samples of the M vs. BA (56 mg/kg) group; Table S11: Differential metabolites identified in serum samples of the C vs. M group; Table S12: Differential metabolites identified in serum samples of the M vs. BA (56 mg/kg) group; Figure S1: Metabolomic workflow flowchart; Figure S2: Blank plasma chromatogram; Figure S3: Spiked plasma chromatogram; Figure S4: Dosed plasma chromatogram; Figure S5: The PCA and OPLS–DA score plot of urine and serum samples in POS and NEG mode; Figure S6: Multi-group difference scatter plots; Figure S7: Volcano plots in POS and NEG mode of urine and serum samples; Figure S8: KEGG enrichment bubble plot of urine and serum; Figure S9: Graph of the results of the topology analysis.
