# Supplementary material for: Pharmacokinetics and Metabolomic Mechanism of the Diuretic Effects of Barbatic Acid from Pyrrosia petiolosa (Christ) Ching
Source: Biology (Basel). 2026 Mar 25;15(7):521. doi: 10.3390/biology15070521 (PMC13072244; doi:10.3390/biology15070521)
Supplement: Supplementary file 1 [file biology-15-00521-s001.zip › biology-4193108-supplementary.pdf]

Table S1. Precision and accuracy of the lower limit of quantitation for BA (n=6)

Concentration (ng/ml)	Intra-day			Inter-day		
	Measured concentration Mean±SD	Accuracy RE%	Precision RSD%	Measured concentration Mean±SD	Accuracy RE%	Precision RSD%
0.125	0.131±0.02	-4.58	12.96	0.121±0.01	3.31	9.35

Table S2. Precision and accuracy of BA in rat plasma (n=6)

QC sample concentrations (ng/ml)	Intra-day			Inter-day		
	Determined concentration Mean±SD	Accuracy RE(%)	Precision RSD(%)	Determined concentration Mean±SD	Accuracy RE(%)	Precision RSD(%)
2.5	2.47±0.29	1.21	11.86	2.32±0.08	7.76	3.47
200	203.29±4.44	-1.48	2.18	198±11.51	1.01	5.80
2000	2001.32±69.52	-0.06	3.47	2027.91±67.35	-1.38	3.32

Table S3 Recovery Rate and Matrix Effect of Bate Acid in Plasma (n=6)

QC sample concentrations (ng/ml)	Recovery Rate(%) Mean±SD	Precision RSD(%)	Matrix Effect(%) Mean±SD	Precision RSD(%)
2.5	93.81±3.12	5.73	93.01±6.39	2.89
200	95.24±5.47	9.63	90.89±3.80	5.43
2000	97.33±8.39	7.25	95.49±5.06	1.20

Table S4 Stability of Barbatic acid in the rat plasma.

Conditions of inspection	Concentration (ng/mL)	Mean of measured concentrations (Mean±SD)	Precision RE(%)
Short-term stability (25℃，4 h)	2.5	2.42±0.28	3.31
	2000	2093.53±91.24	-4.47
Long-term stability (4℃，24 h)	2.5	2.84±0.16	-11.97
	2000	2096.79±93.42	-4.62

(-20℃，15 day)	2.5	2.38±0.10	5.04
	2000	2026.56±52.03	-1.32
Freeze-thaw(%)	2.5	2.19±0.52	14.16
	2000	2014.66±103.78	-0.73

Table S5. Total urine volume (mL) over 6 consecutive hours ($\bar{x}\pm s$，$n=6$)

Groups	Number	Total urine volume (mL)
Control	6	10.80±2.55
Model	6	7.60±1.70*
Positive	6	14.93±2.65##
BA (28 mg/kg)	6	11.67±1.19##
BA (56 mg/kg)	6	11.33±4.31##

Note: Compared with control,*$P<0.05$; Compared with model, ##$P<0.01$

Table S6. Urine volume (mL) at different time points after administration ($\bar{x}\pm s$，$n=6$)

Groups	1 h	2 h	3 h	4 h	5 h	6 h
Control	0.92±0.71	1.15±1.13	1.67±1.31	2.80±1.21	0.63±0.53	1.00±0.23
Model	1.12±0.98	1.02±0.67	2.07±0.95	1.13±0.27	1.27±0.72	1.03±0.26
Positive	4.15±2.80**	3.52±0.86**	3.37±1.08	1.68±0.85	1.47±0.88	1.12±0.42
BA (28 mg/kg)	2.65±1.28*	2.05±0.80*	2.55±1.34	1.53±1.59	1.57±0.62	1.32±0.76
BA (56 mg/kg)	3.13±1.56*	2.33±1.00*	2.15±0.57	1.10±0.61	1.32±0.87	1.30±0.78

Note: Compared with model,*$P<0.05$, **$P<0.01$

Table S7. Effects of BA on urinary Na^+, K^+, and Cl^- excretion in water-loaded rats over 6 h ($\bar{x}\pm s$，$n=6$)

Groups	Na^+(mmol)	K^+(mmol)	Cl^-(mmol)
Control	8.64±1.55	13.79±0.35	32.26±0.05
Model	68.12±22.94**	91.46±1.49**	89.41±5.95**
Positive	109.11±10.07#	92.00±27.60	110.585±12.99#
BA (28 mg/kg)	89.42±18.07#	105.99±21.80#	105.43±17.20#

BA (56 mg/kg)	97.79±13.20$^{\#}$	105.28±18.37$^{\#}$	115.24±17.54$^{\#\#}$

Note: Compared with control, **P＜0.01; Compared with model, #P＜0.05；##P＜0.01 (one-tailed Dunnett's test); two-sided 95% confidence intervals are provided in Table S4.

Table S8 Detailed effect sizes and confidence intervals for all comparisons

Comparison	Ion	Mean difference (mmol)	95% CI for difference	Cohen's d	95% CI for d
Control vs Model	Na+	59.48	[38.58, 80.38]	3.66	[1.70, 5.62]
	K+	77.67	[76.28, 79.06]	71.78	[40.30, 103.26]
	CI-	57.15	[51.74, 62.56]	13.58	[7.52, 19.64]
Model vs Positive	Na+	40.99	[18.21, 63.77]	2.31	[0.80, 3.83]
	K+	0.54	[-24.60, 25.68]	0.03	[-1.10, 1.16]
	CI-	21.18	[8.18, 34.18]	2.10	[0.64, 3.55]
Model vs BA(28 mg/kg)	Na+	21.30	[-5.25, 47.85]	1.03	[-0.19, 2.25]
	K+	14.53	[-5.35, 34.41]	0.94	[-0.26, 2.14]
	CI-	16.02	[-0.53, 32.57]	1.25	[-0.01, 2.50]
Model vs BA(56 mg/kg)	Na+	29.67	[5.60, 53.74]	1.59	[0.26, 2.91]
	K+	13.82	[-2.94, 30.58]	1.06	[-0.16, 2.28]
	CI-	25.83	[8.98, 42.68]	1.97	[0.55, 3.40]

Table S9. Differential metabolites identified in urine samples of the C vs M group

Number	Compound	formula	FC	P	VIP	Model
1	Hippuric acid	$C_9H_9NO_3$	2.2844	0.0111	8.3626	POS
2	Nitrosoheptamethyleneimine	$C_7H_{14}N_2O$	0.6662	0.0465	7.1486	POS
3	Spermidine	$C_7H_{19}N_3$	1.7692	0.0299	4.7888	POS
4	Nicotinamide	$C_6H_6N_2O$	1.6598	0.0004	4.6059	POS
5	N-Acetyl-L-glutamine	$C_7H_{12}N_2O_4$	0.5581	0.0474	3.6666	POS
6	2-[2-oxo-2-(2-pyridylamino) ethoxy]acetic acid	$C_9H_{10}N_2O_4$	1.6246	0.0059	3.1320	POS
7	3,7-Dimethyluric acid	$C_7H_8N_4O_3$	0.6325	0.0095	2.9837	POS
8	o-Cresol	C_7H_8O	1.6816	0.0009	2.9151	POS
9	Cinnamoylglycine	$C_{11}H_{11}NO_3$	1.4903	0.0008	2.8862	POS
10	Dihydrothymine	$C_5H_8N_2O_2$	1.1655	0.0102	2.8736	POS
11	L-(-)-Methionine	$C_5H_{11}NO_2S$	2.2749	0.0003	2.5810	POS
12	Riboflavin	$C_{17}H_{20}N_4O_6$	0.9859	0.0034	2.0670	POS
13	2-Oxindole	C_8H_7NO	1.7742	0.0409	1.9517	POS

14	N-Acetylneuraminic acid	$C_{11}H_{19}NO_9$	0.5048	0.0282	1.9185	POS
15	Diethyl maleate	$C_8H_{12}O_4$	1.9374	0.0003	1.8755	POS
16	D-(-)-Glutamine	$C_5H_{10}N_2O_3$	1.2346	0.0439	1.6451	POS
17	3-(4-hydroxy-3-methoxyphenyl)propanoic acid	$C_{10}H_{12}O_4$	1.2868	0.0021	1.4264	POS
18	3-oxoindane-1-carboxylic acid	$C_{10}H_8O_3$	2.0892	0.0000	1.2811	POS
19	S-Adenosylmethionine	$C_{15}H_{22}N_6O_5S$	2.0139	0.0010	1.2796	POS
20	Rufloxacin	$C_{17}H_{18}FN_3O_3S$	2.1738	0.0001	1.1987	POS
21	D-(+)-Pyroglutamic Acid	$C_5H_7NO_3$	1.0711	0.0000	1.1490	POS
22	indoline-2-carboxylic acid	$C_9H_9NO_2$	0.2889	0.0076	1.1067	POS
23	TAG (16:2-21:5-22:5)	$C_{62}H_{96}O_6$	4.8352	0.0016	1.0557	POS
24	5-Aminosalicylic Acid	$C_7H_7NO_3$	-0.4412	0.0243	3.6683	POS
25	Xanthurenic acid	$C_{10}H_7NO_4$	-1.2514	0.0144	3.3667	POS
26	3-Methylhistamine	$C_6H_{11}N_3$	-1.4190	0.0114	3.0464	POS
27	Nicotinic Acid	$C_6H_5NO_2$	-0.6271	0.0040	2.9513	POS
28	Isorhapontigenin	$C_{15}H_{14}O_4$	-0.6024	0.0145	2.7623	POS
29	2-(acetyloxy)-3-amino-1-[1,2-di(acetyloxy)ethyl]-3-oxopropyl acetate	$C_{13}H_{19}NO_9$	-1.3997	0.0003	2.7482	POS
30	N-[1-(4-methoxy-2-oxo-2H-pyran-6-yl)-2-methylbutyl]acetamide	$C_{13}H_{19}NO_4$	-0.5821	0.0166	2.7188	POS
31	1-Methyladenine	$C_6H_7N_5$	-0.9010	0.0056	2.5955	POS
32	7-(2-aminophenyl)heptanoic acid	$C_{13}H_{19}NO_2$	-1.7792	0.0109	2.4518	POS
33	Boc-beta-cyano-L-alanine	$C_9H_{14}N_2O_4$	-0.8023	0.0101	2.3277	POS
34	FLK	$C_{21}H_{34}N_4O_4$	-1.8587	0.0195	2.2785	POS
35	Norepinephrine	$C_8H_{11}NO_3$	-3.5687	0.0000	2.2782	POS
36	4-Hydroxymandelonitrile	$C_8H_7NO_2$	-2.1220	0.0053	2.2126	POS
37	5-hydroxy-4-methoxy-5,6-dihydro-2H-pyran-2-one	$C_6H_8O_4$	-0.8707	0.0102	2.2037	POS
38	2,3-Dideoxyuridine	$C_9H_{12}N_2O_4$	-3.3463	0.0088	2.1607	POS
39	N4-Acetylcytidine	$C_{11}H_{15}N_3O_6$	-0.6382	0.0059	2.1272	POS
40	6-Hydroxymelatonin	$C_{13}H_{16}N_2O_3$	-2.8737	0.0012	2.0567	POS
41	Glycylproline	$C_7H_{12}N_2O_3$	-0.5668	0.0041	1.9930	POS
42	5-(2,5-dihydroxyhexyl)oxolan-2-one	$C_{10}H_{18}O_4$	-0.6874	0.0169	1.9123	POS
43	Nicotinuric acid	$C_8H_8N_2O_3$	-0.5602	0.0109	1.7442	POS
44	gamma-Glutamylleucine	$C_{11}H_{20}N_2O_5$	-1.9059	0.0001	1.6617	POS
45	4-[4-(aminocarbonyl)piperidino]-4-oxobut-2-enoic acid	$C_{10}H_{14}N_2O_4$	-0.5790	0.0002	1.5616	POS
46	Diaminopimelic acid	$C_7H_{14}N_2O_4$	-0.6245	0.0239	1.5589	POS

47	10-Camphorsulfonic acid	$C_{10}H_{16}O_4S$	-0.9226	0.0044	1.5470	POS
48	Propionyl-L-carnitine	$C_{10}H_{19}NO_4$	-1.5787	0.0084	1.4540	POS
49	Flavanone	$C_{15}H_{12}O_2$	-1.1631	0.0000	1.3701	POS
50	1-(2-furyl)-3,3-di(methylthio)prop-2-en-1-one	$C_9H_{10}O_2S_2$	-1.6443	0.0094	1.3321	POS
51	7-Methylguanosine	$C_{11}H_{15}N_5O_5$	-0.9875	0.0029	1.3289	POS
52	3-methyl-5-oxo-5-(4-toluidin o)pentanoic acid	$C_{13}H_{17}NO_3$	-0.6699	0.0098	1.2455	POS
53	Citrinin	$C_{13}H_{14}O_5$	-4.3495	0.0383	1.2082	POS
54	(S)-Equol	$C_{15}H_{14}O_3$	-0.4549	0.0242	1.1241	POS
55	N-Acetylhistamine	$C_7H_{11}N_3O$	-0.3828	0.0461	1.0095	POS
56	Uric acid	$C_5H_4N_4O_3$	1.0854	0.0000	4.6646	NEG
57	Phenobarbital	$C_{12}H_{12}N_2O_3$	0.8159	0.0310	2.8309	NEG
58	methadone-d9	$C_{21}H_{27}NO$	3.8597	0.0018	2.6293	NEG
59	Hexanoylglycine	$C_8H_{15}NO_3$	1.1381	0.0236	2.4573	NEG
60	L-Adrenaline	$C_9H_{13}NO_3$	0.5138	0.0336	1.9671	NEG
61	3-amino-1H-pyrazolo[4,3-c] pyridine-4,6-diol	$C_6H_6N_4O_2$	0.3634	0.0248	1.6279	NEG
62	2,6-Dihydroxypurine	$C_5H_4N_4O_2$	0.6931	0.0131	1.6010	NEG
63	D-Threose	$C_4H_8O_4$	0.9288	0.0002	1.0692	NEG
64	2-{2-[2,5-di(methoxycarbon yl)anilino]-2-oxoethoxy}acet ic acid	$C_{14}H_{15}NO_8$	1.5585	0.0454	1.0131	NEG
65	Sulfamethoxazole hydroxylamine	$C_{10}H_{11}N_3O_4S$	2.1665	0.0014	1.0117	NEG
66	DL-Dihydroorotic acid	$C_5H_6N_2O_4$	0.7619	0.0021	1.0026	NEG
67	Isocitric acid	$C_6H_8O_7$	-1.2520	0.0034	12.9242	NEG
68	Suberic acid	$C_8H_{14}O_4$	-0.8345	0.0338	2.0851	NEG
69	cis-Aconitic acid	$C_6H_6O_6$	-1.0482	0.0437	1.9078	NEG
70	D-伪-Hydroxyglutaric acid	$C_5H_8O_5$	-1.1225	0.0017	1.7748	NEG
71	Glutaric acid	$C_5H_8O_4$	-1.7961	0.0033	1.6941	NEG
72	2-Isopropylmalic acid	$C_7H_{12}O_5$	-1.5692	0.0355	1.5464	NEG
73	2-Oxoglutaric acid	$C_5H_6O_5$	-1.1724	0.0378	1.2888	NEG
74	2-Hydroxy-2-methylbutanedi oic acid	$C_5H_8O_5$	-1.0226	0.0352	1.1673	NEG
75	N-Acetyl-aspartic acid	$C_6H_9NO_5$	-1.0744	0.0266	1.0285	NEG

Note: (FLK" is an unidentified metabolite that was significantly altered (VIP > 1, $p < 0.05$) but could not be matched to any known compound in the databases. It is reported based on its accurate mass ($[M+H]^+$ at m/z 204.1343, retention time 1.33 min) and molecular formula $C_{21}H_{34}N_4O_4$; its biological interpretation is currently limited and awaits future identification)

Table S10. Differential metabolites identified in urine samples of the M vs BA (56mg/kg) group

Number	Compound	formula	FC	P	VIP	Mode 1

1	Choline	$C_5H_{13}NO$	1.1822	0.0001	4.6426	POS
2	Hexadecanamide	$C_{16}H_{33}NO$	0.4572	0.0361	4.1971	POS
3	Monobutyl phthalate	$C_{12}H_{14}O_4$	4.2974	0.0000	2.5402	POS
4	7-(2-aminophenyl)heptanoic acid	$C_{13}H_{19}NO_2$	1.2320	0.0373	2.1350	POS
5	Nicotinuric acid	$C_8H_8N_2O_3$	0.4770	0.0041	2.0536	POS
6	Oleoyl ethylamide	$C_{20}H_{39}NO$	0.5802	0.0267	2.0308	POS
7	FLK	$C_{21}H_{34}N_4O_4$	1.2831	0.0464	1.9776	POS
8	Urocanic acid	$C_6H_6N_2O_2$	0.8213	0.0058	1.9514	POS
9	Stearamide	$C_{18}H_{37}NO$	0.5339	0.0268	1.8164	POS
10	3-methyl-5-oxo-5-(4-toluidino)pentanoic acid	$C_{13}H_{17}NO_3$	0.9394	0.0437	1.7839	POS
11	2-(acetyloxy)-3-amino-1-[1,2-di(acetyloxy)ethyl]-3-oxopropyl acetate	$C_{13}H_{19}NO_9$	0.4558	0.0144	1.3846	POS
12	N-Acetylhistamine	$C_7H_{11}N_3O$	0.3650	0.0305	1.2335	POS
13	Trigonelline	$C_7H_7NO_2$	-0.9681	0.0368	5.3451	POS
14	N-[1-(4-methoxy-2-oxo-2H-pyran-6-yl)-2-methylbutyl]acetamide	$C_{13}H_{19}NO_4$	-0.3968	0.0009	2.6488	POS
15	Isorhapontigenin	$C_{15}H_{14}O_4$	-0.4004	0.0006	2.6301	POS
16	DGTS (4:0/5:0)	$C_{19}H_{35}NO_7$	-1.6798	0.0293	2.6211	POS
17	Oxymorphone	$C_{17}H_{19}NO_4$	-0.5177	0.0021	1.9221	POS
18	Riboflavin	$C_{17}H_{20}N_4O_6$	-0.6117	0.0289	1.8640	POS
19	1-(4-methoxyphenyl)propane-1,2-diol	$C_{10}H_{14}O_3$	-1.2007	0.0001	1.5086	POS
20	3,5-dimethyl-1-phenyl-1,5-dihydro-4H-pyrazolo[4,3-c]quinolin-4-one	$C_{18}H_{15}N_3O$	-0.6950	0.0292	1.3414	POS
21	S-Adenosylmethionine	$C_{15}H_{22}N_6O_5S$	-1.2296	0.0046	1.2731	POS
22	3-oxoindane-1-carboxylic acid	$C_{10}H_8O_3$	-0.6873	0.0093	1.0020	POS
23	Taurine	$C_2H_7NO_3S$	1.4047	0.0038	11.9805	NEG
24	3-Coumaric acid	$C_9H_8O_3$	7.0153	0.0000	3.4854	NEG
25	cis-Aconitic acid	$C_6H_6O_6$	1.4127	0.0024	2.4614	NEG
26	1,2,3-cyclopropanetricarboxylic acid	$C_6H_6O_6$	1.2108	0.0065	2.4261	NEG
27	Homovanillic acid	$C_9H_{10}O_4$	5.9121	0.0003	1.6100	NEG
28	2-Hydroxy-2-methylbutanedioic acid	$C_5H_8O_5$	1.3332	0.0022	1.5038	NEG
29	Asaraldehyde	$C_{10}H_{12}O_4$	6.3952	0.0035	1.0318	NEG
30	Citric acid	$C_6H_8O_7$	-1.3851	0.0223	9.6677	NEG
31	Uric acid	$C_5H_4N_4O_3$	-0.5698	0.0055	2.9518	NEG
32	2-Furoic acid	$C_5H_4O_3$	-1.6899	0.0473	2.3708	NEG

33	trans-Aconitic acid	$C_6H_6O_6$	-1.2790	0.0268	2.2749	NEG
34	mesaconic acid	$C_5H_6O_4$	-1.2236	0.0128	1.3585	NEG

Note: (FLK" is an unidentified metabolite that was significantly altered (VIP > 1, p < 0.05) but could not be matched to any known compound in the databases. It is reported based on its accurate mass ($[M+H]^+$ at m/z 204.1343, retention time 1.33 min) and molecular formula $C_{21}H_{34}N_4O_4$; its biological interpretation is currently limited and awaits future identification)

Table S11. Differential metabolites identified in serum samples of the C vs M group

Number	Compound	formula	FC	P	VIP	Model
1	ILK	$C_{18}H_{36}N_4O_4$	2.3808	0.0167	4.7241	POS
2	MAG (18:3)	$C_{21}H_{36}O_4$	2.4706	0.0144	3.9077	POS
3	Creatine	$C_4H_9N_3O_2$	0.5229	0.0350	3.4921	POS
4	ACar 18:1	$C_{25}H_{48}NO_4$	0.5375	0.0008	2.0648	POS
5	APK	$C_{14}H_{26}N_4O_4$	0.3785	0.0114	1.8442	POS
6	Palmitoylcarnitine	$C_{23}H_{45}NO_4$	0.3625	0.0044	1.6637	POS
7	TAG (12:2-16:5-18:1)	$C_{49}H_{78}O_6$	0.2247	0.0105	1.3282	POS
8	ACar 18:2	$C_{25}H_{46}NO_4$	0.5022	0.0029	1.2765	POS
9	(囟)13-HpODE	$C_{18}H_{32}O_4$	1.7785	0.0292	1.1945	POS
10	Oleic acid	$C_{18}H_{34}O_2$	0.4806	0.0359	1.1633	POS
11	SNH	$C_{13}H_{20}N_6O_6$	0.7291	0.0467	1.0285	POS
12	PC (18:1/18:1)	$C_{44}H_{84}NO_8P$	-0.1820	0.0425	8.7089	POS
13	PC (20:2/20:3)	$C_{48}H_{86}NO_8P$	-0.4531	0.0087	3.5892	POS
14	1-Methyladenine	$C_6H_7N_5$	-0.2681	0.0064	1.6192	POS
15	trans-4-Hydroxy-L-proline	$C_5H_9NO_3$	-0.2603	0.0074	1.6056	POS
16	PC (18:0/19:2)	$C_{45}H_{86}NO_8P$	-0.4197	0.0367	1.0278	POS
17	Cholic acid	$C_{24}H_{40}O_5$	2.2053	0.0135	8.5201	NEG
18	Deoxycholic acid	$C_{24}H_{40}O_4$	1.2490	0.0038	6.9169	NEG
19	7-Ketolithocholic acid	$C_{24}H_{38}O_4$	1.1701	0.0195	2.5806	NEG
20	(囟)12(13)-DiHOME	$C_{18}H_{34}O_4$	0.9279	0.0409	2.3239	NEG
21	Sodium cholate	$C_{24}H_{39}NaO_5$	1.5629	0.0249	2.2824	NEG
22	3-Oxo-7alpha,12alpha-hydroxy-5beta-cholanoic acid	$C_{24}H_{38}O_5$	2.3150	0.0235	1.6694	NEG
23	2-Hydroxyvaleric acid	$C_5H_{10}O_3$	0.6879	0.0322	1.3222	NEG
24	Dodecanedioic acid	$C_{12}H_{22}O_4$	0.8469	0.0036	1.2537	NEG
25	LPC 18:2	$C_{26}H_{50}NO_7P$	-0.1815	0.0477	6.2412	NEG
26	LPC 20:2	$C_{28}H_{54}NO_7P$	-0.5489	0.0125	1.9187	NEG
27	LPS 20:0	$C_{26}H_{52}NO_9P$	-5.0310	0.0107	1.7888	NEG
28	2-({[4-(6-methyl-1,3-benzothiazol-2-yl)phenyl]imino}methyl)phenol	$C_{21}H_{16}N_2OS$	-0.4032	0.0430	1.4571	NEG
29	Glu-Gln	$C_{10}H_{17}N_3O_6$	-0.4370	0.0459	1.1235	NEG

Note: (It is important to note that a number of differentially abundant features (VIP > 1, p < 0.05) remain unidentified, as they could not be matched to any known compound in the current databases. Therefore, their biological interpretation is currently constrained, and their potential

biological roles remain to be elucidated in future studies.)

Table S12. Differential metabolites identified in serum samples of the M vs BA (56mg/kg) group

Number	Compound	formula	FC	P	VIP	Model
1	Oleamide	$C_{18}H_{35}NO$	0.3231	0.0159	9.0498	POS
2	Arachidonoyl amide	$C_{20}H_{33}NO$	0.6091	0.0357	4.5380	POS
3	Hexadecanamide	$C_{16}H_{33}NO$	0.4108	0.0223	4.1578	POS
4	4-methoxy-6-(prop-2-en-1-yl)-2H-1,3-benzodioxole	$C_{11}H_{12}O_3$	8.6351	0.0000	3.0986	POS
5	3-hydroxy-3,4-bis[(4-hydroxy-3-methoxyphenyl)methyl]oxolan-2-one	$C_{20}H_{22}O_7$	7.0232	0.0000	2.7449	POS
6	DL-Arginine	$C_6H_{14}N_4O_2$	0.2682	0.0207	2.2034	POS
7	L-Threonine	$C_4H_9NO_3$	0.3844	0.0475	2.0682	POS
8	PC (20:3/22:6)	$C_{50}H_{82}NO_8P$	0.4812	0.0472	1.7664	POS
9	Pyridoxamine	$C_8H_{12}N_2O_2$	0.5450	0.0368	1.5841	POS
10	5,6-dimethyl-4-oxo-4H-pyran-2-carboxylic acid	$C_8H_8O_4$	0.8853	0.0022	1.2107	POS
11	Taurine	$C_2H_7NO_3S$	0.5450	0.0389	1.2012	POS
12	1-Pyrenol	$C_{16}H_{10}O$	0.2758	0.0381	1.0811	POS
13	PC (18:2/18:3)	$C_{44}H_{78}NO_8P$	-0.8470	0.0175	3.0312	POS
14	Corticosterone	$C_{21}H_{30}O_4$	-0.4607	0.0026	3.0029	POS
15	SM (d18:2/16:0)	$C_{39}H_{77}N_2O_6P$	-0.4787	0.0405	2.7200	POS
16	SM (d19:0/14:1)	$C_{38}H_{77}N_2O_6P$	-0.6118	0.0022	1.8962	POS
17	PC (20:4/22:6)	$C_{50}H_{80}NO_8P$	-0.6392	0.0326	1.2629	POS
18	Hippuric acid	$C_9H_9NO_3$	-0.9487	0.0260	1.0699	POS
19	3-Coumaric acid	$C_9H_8O_3$	8.3485	0.0000	6.1780	NEG
20	Chenodeoxycholic Acid	$C_{24}H_{40}O_4$	1.5665	0.0281	5.3767	NEG
21	Homovanillic acid	$C_9H_{10}O_4$	5.0117	0.0000	4.8534	NEG
22	Chlorogenic acid	$C_{16}H_{18}O_9$	8.3032	0.0000	1.7030	NEG
23	Taurolithocholic acid	$C_{26}H_{45}NO_5S$	1.3028	0.0172	1.2447	NEG
24	Tyrosol	$C_8H_{10}O_2$	4.9429	0.0063	1.1670	NEG
25	Prostaglandin K1	$C_{20}H_{32}O_5$	0.6967	0.0197	1.0089	NEG
26	Glycocholic acid	$C_{26}H_{43}NO_6$	-1.9718	0.0159	6.2626	NEG
27	LPS 18:2	$C_{24}H_{44}NO_9P$	-0.6099	0.0351	1.6026	NEG
28	LPE 18:1	$C_{23}H_{46}NO_7P$	-0.3541	0.0297	1.5556	NEG
29	2-Hydroxyvaleric acid	$C_5H_{10}O_3$	-0.6165	0.0403	1.5414	NEG
30	Dodecanedioic acid	$C_{12}H_{22}O_4$	-0.9811	0.0019	1.3504	NEG
31	Phenylacetaldehyde	C_8H_8O	-1.0386	0.0179	1.3359	NEG
32	Glycochenodeoxycholic Acid (sodium salt)	$C_{26}H_{43}NO_5$	-0.8469	0.0291	1.3142	NEG
33	15-OxoEDE	$C_{20}H_{34}O_3$	-0.6232	0.0451	1.0656	NEG
34	3-Methyladipic acid	$C_7H_{12}O_4$	-0.9330	0.0406	1.0272	NEG

Note: (It is important to note that a number of differentially abundant features (VIP > 1, p < 0.05)

remain unidentified, as they could not be matched to any known compound in the current databases. Therefore, their biological interpretation is currently constrained, and their potential biological roles remain to be elucidated in future studies.)

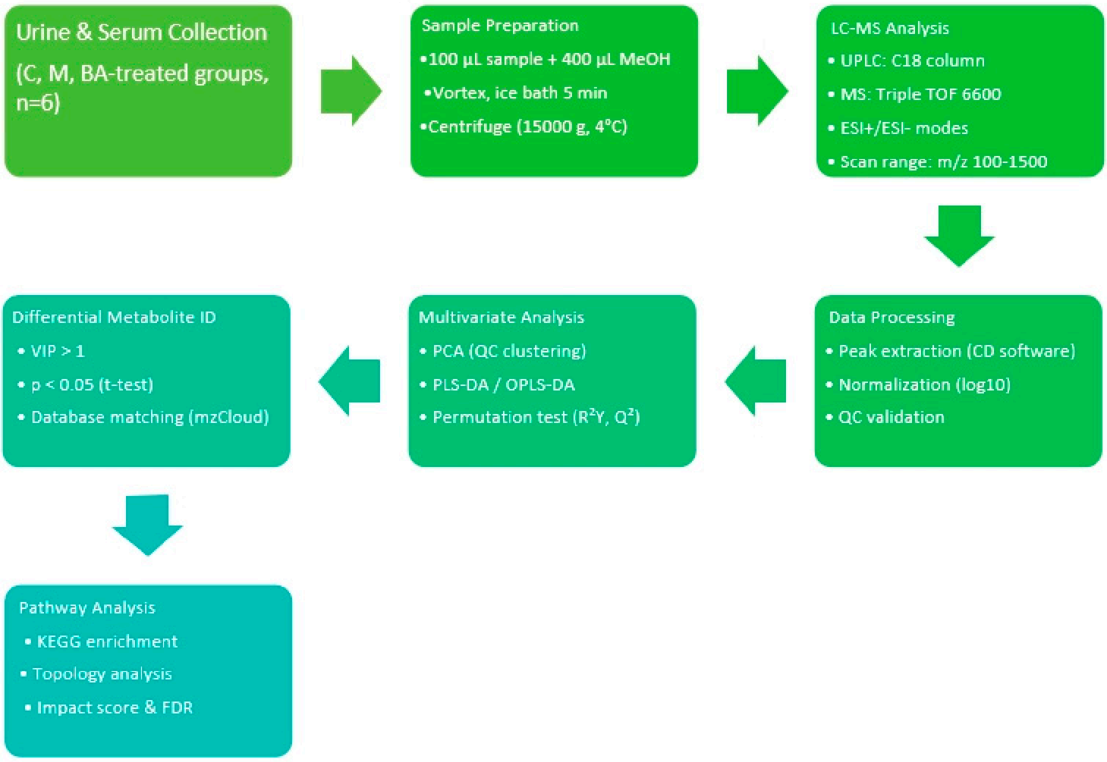

Figure S1 Metabolomic workflow flowchart

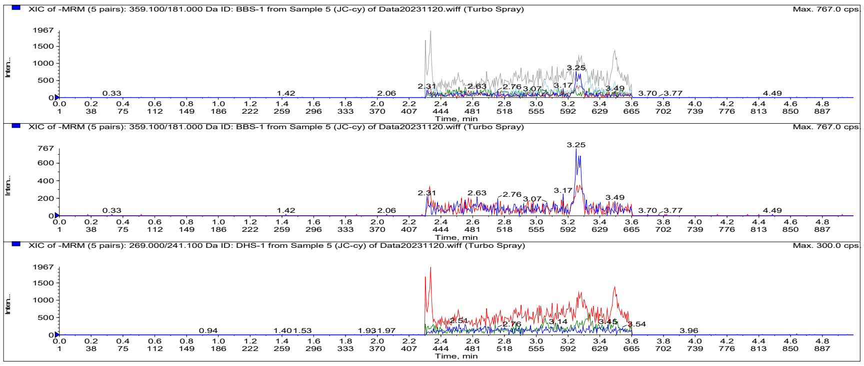

Figure S2. Blank plasma chromatogram

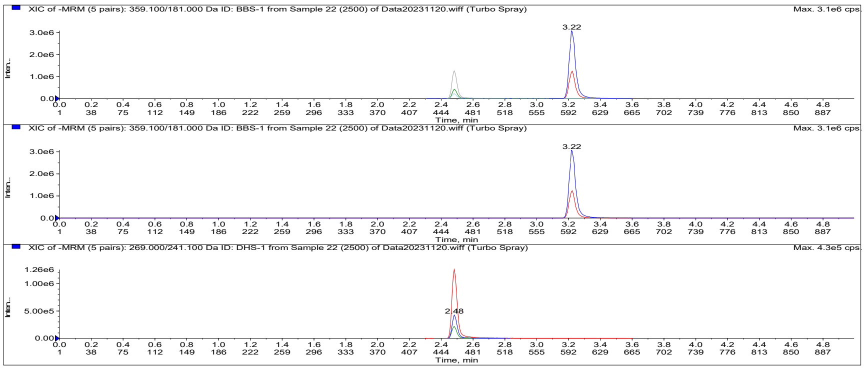

Figure S3. Spiked plasma chromatogram

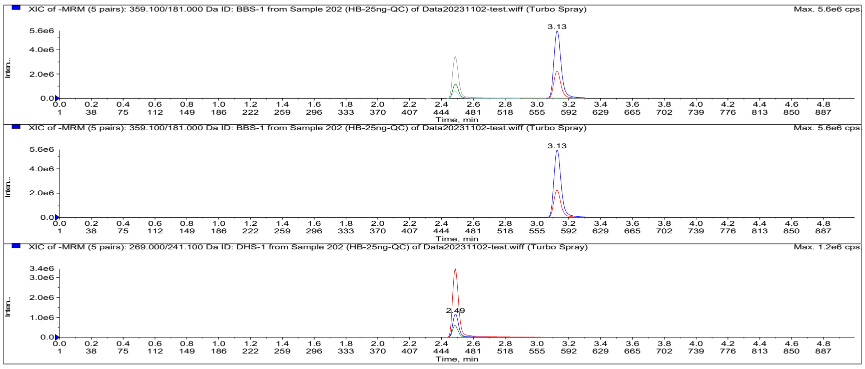

Figure S4. Dosed plasma chromatogram

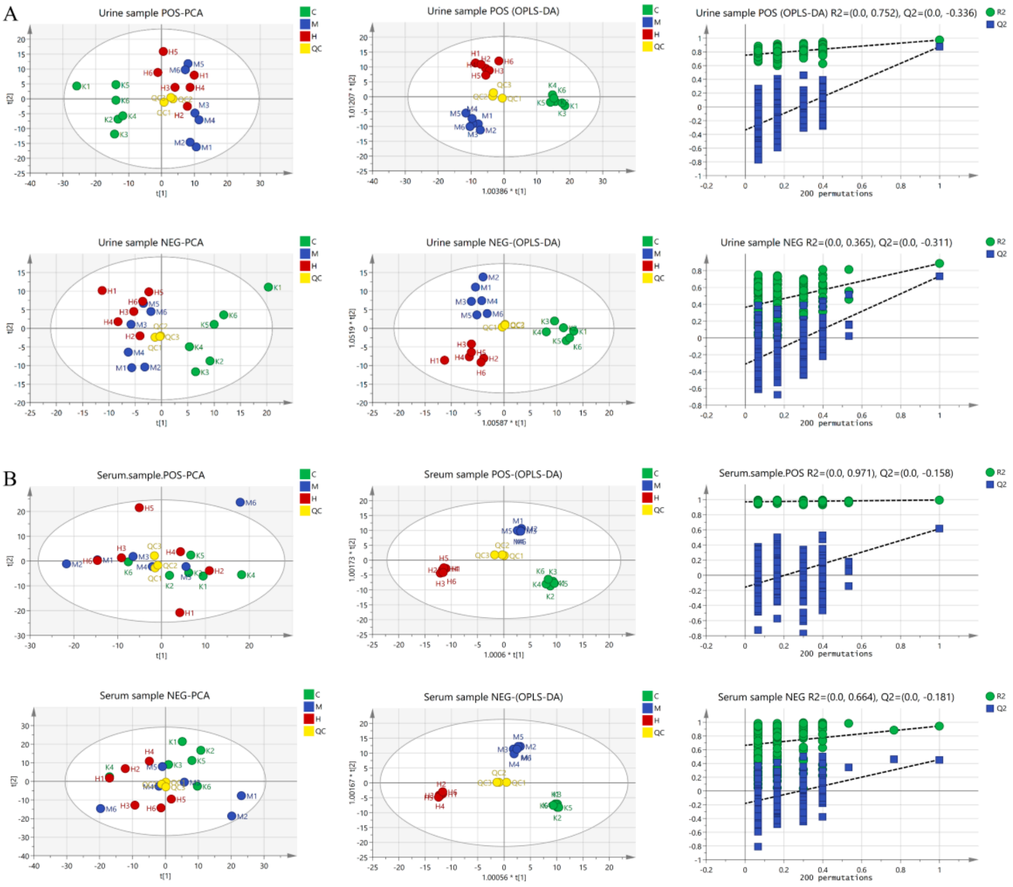

Figure S5. The PCA and OPLS－DA score plot of urine and serum samples in POS and NEG

mode. (A is the PCA, OPLS-DA, and Permutation test diagram of the urine metabolome; B is the PCA, OPLS-DA, and Permutation test diagram of the blood metabolome.)

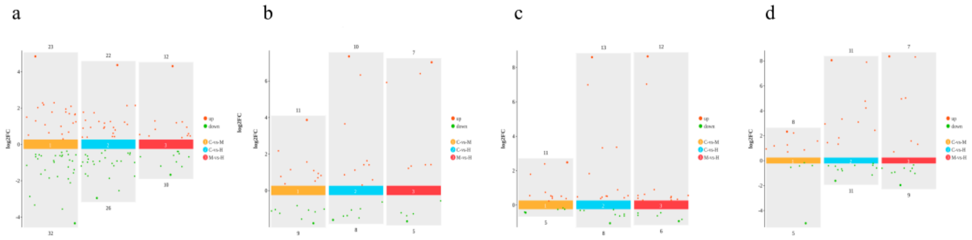

Figure S6**.** Multi-group difference scatter plots (a. Urine, POS mode b. Urine, NEG mode c. Serum, POS mode d. Serum, NEG mode)

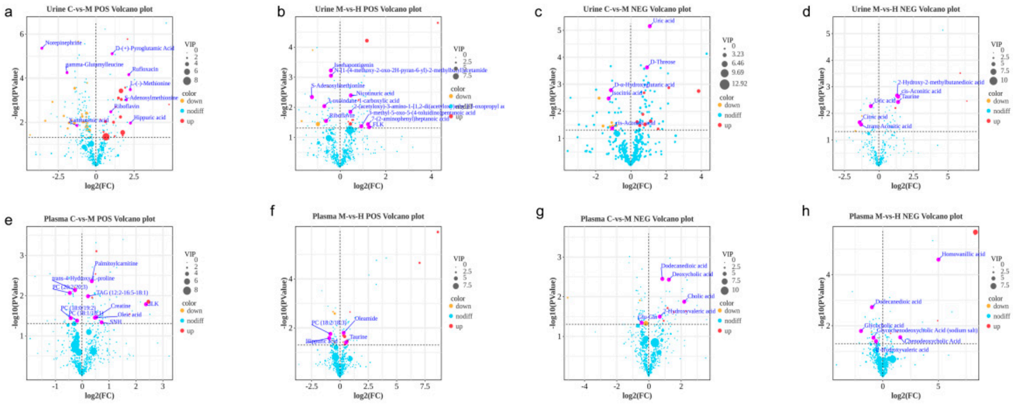

Figure S7. Volcano plots in POS and NEG mode of urine and serum samples

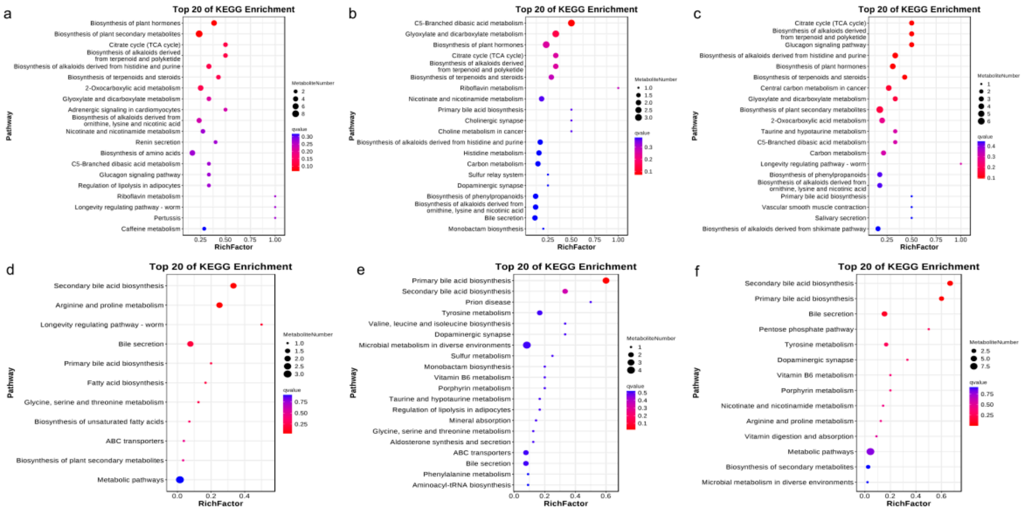

Figure S8. KEGG enrichment bubble plot of urine and serum: the top 20 pathways with the smallest Q-value are used to make the plot, the vertical coordinate is the pathway, the horizontal coordinate is the enrichment factor (the number of differential metabolites in the pathway divided by all the quantities in the pathway), and the size indicates how many quantities there are, the redder the colour the smaller the Q-value. a. Urine C-vs-M; b. Urine M-vs-BA (56mg/kg); c. Urine C-vs-BA (56mg/kg). d. Serum C-vs-M; e. Serum M-vs-BA (56mg/kg); f. Serum C-vs-BA (56mg/kg)

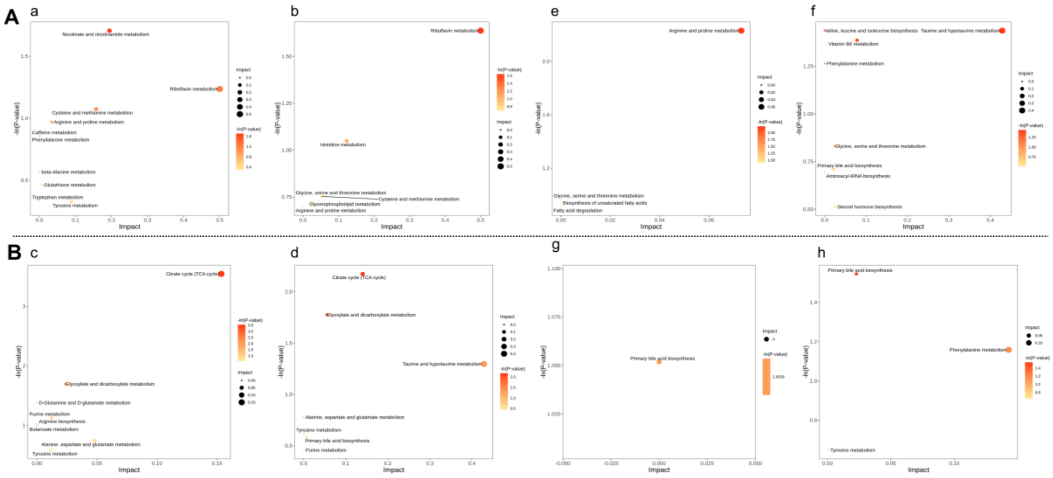

Figure S9. Graph of the results of the topology analysis (A means POS Mode. a. Urine C-vs-M; b. Urine M-vs-BA (56mg/kg); e. Serum C-vs-M; f. Serum M-vs-BA (56mg/kg); B means NEG Mode. c. Urine C-vs-M; d. Urine M-vs-BA (56mg/kg); g. Serum C-vs-M; h. Serum M-vs-BA (56mg/kg).)
